# A New Species *Bussabanomyces oryzae* Isolated from Rice and Beneficial Application in Rice Seedling

**DOI:** 10.3390/jof12030222

**Published:** 2026-03-19

**Authors:** Mengdi Dai, Xiangfeng Tan, Ziran Ye, Yu Luo, Xuting Chen, Bojun Li, Dedong Kong

**Affiliations:** Institute of Digital Agriculture, Zhejiang Academy of Agricultural Sciences, Hangzhou 310021, China; daimd@zaas.ac.cn (M.D.); tanxf@zaas.ac.cn (X.T.); yezr@zaas.ac.cn (Z.Y.); luoyu@zaas.ac.cn (Y.L.); nar_c2004@163.com (X.C.); hzhtcy@126.com (B.L.)

**Keywords:** endophyte, rice seedling, interaction, plant growth promoting, resistance

## Abstract

Endophytes are a type of microorganism that lives in harmony with plants, playing a significant role in promoting the growth of the host and enhancing the host’s stress resistance. Understanding the ecological functions of root endophytic fungi and screening functional strains can effectively alleviate the stress conditions of crops. In this study, endophyte 1R13 was isolated from the roots of rice. Through morphological observation and five-gene combined phylogenetic analysis, it was identified as *Bussabanomyces oryzae *(*B. oryzae*), which was proposed as a new species, *Bussabanomyces oryzae* nov. The colonization pattern of *B. oryzae* was mainly through invasion of the rice roots, entering the epidermal cells and then the cortical cells, and finally reaching the vascular bundle cells. In the co-culture assays with rice, *B. oryzae* can promote the growth of rice, increasing its growth volume by approximately 23% and its fresh weight by 52%. Meanwhile, it could enhance the stress resistance of rice, mainly manifested as increasing the ability of rice leaves to resist rice blast and improving the survival rate of transplanted seedlings in the field.

## 1. Introduction

At present, most of the typical functional microbes are mainly rhizobia and mycorrhizal fungi [1]. As “plant nutrition type” regulators, they can promote the growth of crops and increase yields [2]. However, the effects of rhizobia and mycorrhizal fungi in improving the host’s stress resistance are not ideal [3]. Endophytes are a type of fungi that can colonize within plants without causing fundamental harm to them; at least part of their life cycle is within healthy plant tissues, colonizing and absorbing nutrients from the host [4]. However, unlike pathogenic fungi, endophytes do not cause significant harm to the host. It can maintain a balanced relationship with the host internally [5,6]. During the interaction between endophytic fungi and plants, many beneficial biological relationships have been demonstrated, such as promoting the growth of the host, increasing the host’s yield, and enhancing the host’s stress resistance (disease resistance and heavy metal stress) [4,7,8]. Endophytes are also natural sources of active substances and can produce active metabolic products with antibacterial and bactericidal properties [9].

Endophytes in rice have been found to be associated with various parts of the rice plant, including roots, stems, leaves, seeds, ovules, and even meristems. Among them, roots were the largest source of endophytic bacteria [10,11], followed by leaves. Seeds played a crucial role in the spread of endophytic bacteria in different plants through vertical (parental) and horizontal (environmental characteristics) methods [12]. *Gaeumannomyces graminis* I-1 was found in the roots of rice and had the effect of promoting plant growth [13]. *Pyricularia sacc* was present in the roots and seeds of rice and had antipathogenic activity [14]. *Pseudophhialophora oryzae* could promote rice growth with the increase in fresh weight and chlorophyll content [15]. *Falciphora oryzae* induced systemic resistance in plants and enhanced the tolerance of rice to Cd [16]. These fungi all belong to the Magnaporthaceae family. Bussabanomyces is a genus under the Magnaporthaceae family, and it has only one species, *Bussabanomyces longisporus*. *B. longisporus* was discovered in the leaves of rice [17]. At present, the observations on this family have been limited to morphological studies, and the specific functions of it have not been deeply explored.

Based on the significant potential of endophytic fungi in promoting plant growth and enhancing stress resistance and considering the important position of the Magnaporthaceae family in plant pathology and symbiosis, this study focuses on a newly isolated endophytic strain from rice roots, *Bussabanomyces oryzae*. This study aims to clarify the taxonomic status of this strain through phylogenetic analysis and to reveal its specific colonization pattern in rice roots via GFP fluorescence labeling technology. On this basis, we will further investigate the biological effects of *B. oryzae* on rice growth and its ability to induce disease resistance, attempting to elucidate the potential of this endophytic fungus in enhancing plant stress resistance. The results of this study are intended to provide new microbial resources and a theoretical basis for the development of multifunctional biopesticides and biofertilizers.

## 2. Materials and Methods

### 2.1. Sample Collection and Culture

In June 2024, rice samples were collected in Shangyu District, Shaoxing City, Zhejiang Province, China (N 29°49′–29°53′, E 121°3′–121°7′). When collecting samples, uproot the plants, place the plant samples in self-sealing sterile bags, then put them in an insulated box and store them in ice packs. The samples were brought back to the laboratory for separation and culture within 48 h. The roots of the rice plants were rinsed to remove soil particles and attachments. The root tissue was cut into 0.5 cm segments and separated on the MEA medium containing 50 mg/L chloramphenicol. The strain was cultured in the dark at 25 °C. After the hyphae grew from the edge of the tissue incision, it was carefully picked out with a sterile toothpick and transferred to the PDA medium for purification at 25 °C.

### 2.2. DNA Extraction and Phylogenetic Analysis

DNA extraction was carried out through the fungal DNA extraction kit (Qiagen, Hilden, Germany). Referring to the method of Zhang et al. [18], the largest subunit of RNA polymerase II (RPB1) and the translation Elongation Factor 1-α gene (TEF1), internal transcribed spacer (ITS1), large subunit (LSU), and small subunit (SSU) of ribosomal RNA genes were sequenced and analyzed. Primers are listed in Supplementary Table S1. The obtained sequences were analyzed using BLAST (NCBI) (https://blast.ncbi.nlm.nih.gov/Blast.cgi, aceess date: 4 October 2025) and aligned with reference *Bussabanomyces orzae* strains in CLUSTAL X 2.1 [19]. The isolates and corresponding GenBank accession numbers are provided in Table 1. The alignment was manually refined in GENEDOC [20] to remove extra 5′ and 3′ overlapping regions. DAMBE5 [21] was employed to assess substitution saturation. For phylogenetic reconstruction, we performed Bayesian Inference (BI) using MrBayes v.3.2.7 [22], running 5,000,000 MCMC generations (sampling every 1000 generations). The first 1250 trees (25%) were discarded as burn-in, and the remaining 3750 trees were used to compute posterior probabilities (PPs) in the consensus tree. Maximum Likelihood (ML) analysis was performed using IQ-TREE v.2.3.6 [23], with branch support evaluated via ultrafast bootstrap [24]. The optimal nucleotide substitution model for both BI and ML was selected using jModelTest 2.1.10 [25] under the Akaike Information Criterion (AIC)**.** Finally, p-distances (transitions + transversions) were computed using MEGA 12 [26].

### 2.3. Morphological Observation and Genetic Transformation

For colony observation, mycelial plugs were inoculated on the potato dextrose agar (PDA), malt extract agar (MEA), and water agar (WA) mediums at 25 °C in the dark for 5–7 d. For the observation of mycelial and conidial morphology, fresh mycelial plugs were obtained using an 8 mm diameter puncher and placed in 150 mL potato glucose broth (PDB). The mixture was cultured at 25 °C with a speed of 150 rpm for 3 days. The mycelia and conidia were collected and observed under a microscope (Carl Zeiss, Oberkochen, Germany). At the same time, the fungal mycelium plugs (5 mm × 5 mm) were fixed in 2.5% glutaraldehyde solution at 4 °C overnight. Then, the plugs were rinsed with 0.1 M phosphate buffer solution (pH = 7) for 15 min. The rinsed plugs were placed in 1% OsO_4_ at 25 °C for fixation for 2 h. After fixation, the disks were rinsed with phosphate buffer solution (pH = 7) and dehydrated using graded ethanol. The samples were dried and coated on an HCP-2 critical point dryer (Hitachi, Tokyo, Japan) and then observed under an SU-8010 scanning electron microscope (SEM) (Hitachi, Japan) [27].

The strain 1R13 was incubated in PDB medium for three days. Subsequently, a spore suspension was harvested and adjusted to a concentration of 1 × 10^5^ spores/mL. This suspension was then mixed with an equal volume of an *Agrobacterium tumefaciens* culture containing the PKD3-GFP vector with a benomyl-resistant gene [28]. Transformants were selected on complex medium (CM) supplemented with 400 μg/mL benomyl, 60 μg/mL streptomycin sulfate, and 400 μg/mL cefotaxime. Individual colonies growing on the initial selection plates were subjected to three rounds of single-spore isolation on CM medium also containing 400 μg/mL benomyl, 60 μg/mL streptomycin sulfate, and 400 μg/mL cefotaxime. Fluorescence was visualized using an LSM880 confocal laser scanning microscope (Carl Zeiss, Germany). Prior to inoculating rice plants, a small piece of mycelium from the purified culture was inoculated into LB broth and incubated at 37 °C with shaking for 48 h. No turbidity was observed, confirming that the culture was free of viable Agrobacterium.

### 2.4. Quantification of Fungal Biomass in Roots by qRT-PCR

After 14 days of co-culture with the strain 1R13 labeled with GFP, the roots of the symbiotic organisms were collected, sectioned horizontally and longitudinally, and observed under the LSM880 confocal laser scanning microscope (Carl Zeiss, Germany).

The fungal infection in the roots of rice could be detected by measuring the ratio of fungal/plant DNA (FPDR). The rice seeds were inoculated with 2 mL conidia suspension (5 × 10^5^ conidia/mL) of 1R13 strains and harvested at 5, 10, 15, and 20 days post-inoculation (d.p.i), based on the methods described in a previous study [29]. The root samples were ground in liquid nitrogen. Genomic DNA was extracted from 100 mg of root powder using a Plant DNeasy Kit (Qiagen, Germany) according to the manufacturer’s instructions. For qRT-PCR, 12.5 mL of TB Green (Takara, Osaka, Japan), 0.25 μL of 25 mM OsUbiq-F/R or TEF1-F/R (Supplementary Table S1), and 25 ng of DNA were mixed [30]. The qRT-PCR was performed using a Mastercycler ep realplex Thermal Cycler (Eppendorf, Westbury, NY, USA) with two steps and a melting curve analysis. Each fungal DNA sample was analyzed in triplicate. The assay was repeated at least twice.

### 2.5. Co-Culture and Phenotype Analysis

For the aseptic culture assay, the husks of rice seeds were removed, and the intact grains were selected for germination. For surface sterilization, seeds were sequentially treated with 75% ethanol (5 min) followed by 1% sodium hypochlorite solution (containing 5% active chlorine) for 10 min. After that, the rice seeds were put into MS medium and sealed with a sealing film to prevent contamination. The disinfected seeds were placed in a dark incubator for cultivation for 3 d. The germinated seeds were transplanted into tissue culture flasks (9.6 cm wide and 18.6 cm high) containing 150 mL of 1/2 MS medium, and 10 seedlings were inoculated in each flask. Meanwhile, three fresh endophytic fungal mycelial plugs with a diameter of 8 mm, cultured for 5–7 d, were inoculated into each tissue culture flask. The cultivation conditions were alternating cycles of light exposure at 25 °C for 16 h and darkness at 22 °C for 8 h. Blank blocks were inoculated as a control.

For the pot assay, the endophyte plugs were placed in 150 mL of PDB medium and cultured at 150 rpm at 25 °C for 3 d. Then, the hyphae suspension was added to the soybean cake flour fermentation liquid (0.4% soybean cake powder, 1% maize extract powder, 0.05% magnesium sulfate, 0.1% dipotassium phosphate) and fermented at 25 °C for 7 d. The rice varieties were indica rice CO39 and japonica rice ZH11. The germinated rice seeds mentioned above were planted into planting pots containing substrate soil (30 seeds per pot), and then 200 mL of fermentation liquid was added to each pot. The original liquid of the soybean cake flour fermentation liquid was taken as a control. The fermentation liquid should be irrigated every other week until the rice reaches the three-leaf and one-heart stage.

### 2.6. Rice Phenotype Analysis

When the rice seedling reached the three-leaf and one-heart stage (the control group and the treatment group reached this stage at approximately the same time), the phenotype analysis was conducted. The chlorophyll content was determined by SPAD-502. Plant height was measured as the vertical distance from the soil surface to the apex of the tallest leaf in the canopy. The stem thickness was measured by a vernier caliper. Fresh weight and dry weight were measured by an electronic balance. Fifty rice plants from the control and the treatment groups were selected for measurement. Each experiment was repeated three times.

The rice blast fungus (*Magnaporthe oryzae*) Guy11 was cultured in complete medium (CM) for 10 d, and the spores were collected to prepare a suspension at a concentration of 1 × 10^5^ CFU. Meanwhile, a 0.4% gelatin solution was prepared to increase the adhesion of the spore suspension. After the gelatin solution cooled down, it was mixed with the conidia suspension in equal volumes, and then the mixed solution was evenly sprayed on the co-cultured rice leaves (1 mL/bottle, 2.5 mL/pot). After the sprayed rice was cultivated in the dark at 22 °C for 2 d, it was placed at 25 °C for alternating light and dark cultivation (16 h of light/8 h of dark) for 4 d, and the lesion rate was calculated.

### 2.7. Measurement of Phenotypic and Physiological Indicators of Rice Seedlings in the Field

After the rice seedlings had grown for about 20 d, they were transplanted into the field. A total of 12 planting plots were set up, with 6 replicates in each plot and 1 plot in each replicate. The area of each plot was approximately 70 square meters. After growing in the field for 15 d, 100 rice seedlings were randomly selected from each plot, and the number of dead seedlings was recorded. Six rice seedlings were randomly selected from each repeat. Take a certain amount of plant tissue, wipe off the water and impurities, cut it into pieces, and put it into a mortar. Add liquid nitrogen and grind it into a powder. Then transfer it out. Weigh 0.1 g of the tissue and add 1 mL of the extraction solution, vortex mix and extract for 3–5 min. Centrifuge at 8000× *g*, 4 °C for 10 min. Use the commercial chemical assay kits (Nanjing Jiancheng Bioengineering Institute, Nanjing, China) according to the manufacturer’s instructions to measure the contents of catalase (CAT), peroxidase (POD), and superoxide dismutase (SOD) in the spectrophotometer at wavelengths of 405 nm, 470 nm, and 560 nm, respectively. Each sample was examined in triplicate.

### 2.8. Determination of Defense-Related Gene Expression Levels

Rice samples after co-culture of endophytic fungi and rice were collected, and rice RNA was extracted by the Trizol reagent (Invitrogen, Carlsbad, CA, USA). RNA was reverse-transcribed into cDNA through the reverse transcription kit (Takara, Japan). The expression levels of defense-related genes in rice seedlings were determined by real-time fluorescence quantitative experiments. Finally, the relative expression levels of gene expression were calculated by 2^−ΔΔCt^.

### 2.9. Statistical Analysis

The software GraphPad Prism 7 and SPSS 24.0 software were used for statistical analysis. The data were analyzed using an independent sample *t*-test. The lesion area was assessed by an Axiovision image analyzer.

## 3. Results

### 3.1. Phylogenetic Analysis

**Figure 1:**
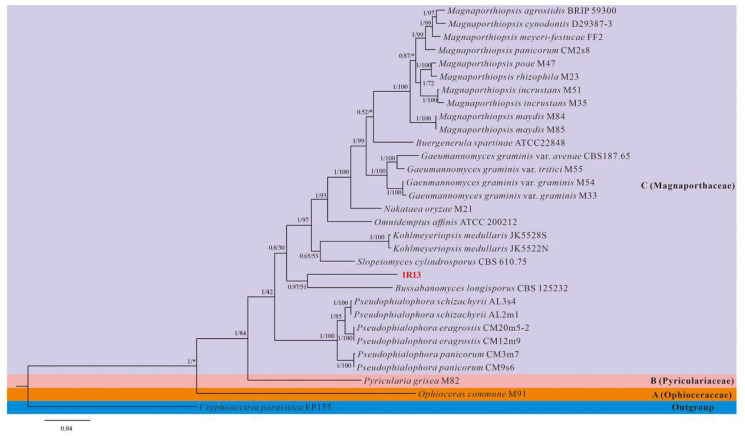
A phylogenetic tree based on the combined ITS-LSU-SSU-TEF1-RPB1 5-gene dataset. The trees’ topological structures use phylogenetic trees constructed by BI methods. The Bar indicates the presence of 0.04 base substitution sites. * indicates the nodes in the phylogenetic tree where the topological structures reconstructed from the BI method and the ML method are inconsistent.

We blasted the five gene sequences of strain 1R13 on the NCBI website (Supplementary Table S2). The results showed that the most similar strains were all from the genus in Magnaporthaceae but were not identified to the species level. To determine the taxonomic status of strain 1R13, we constructed a phylogenetic tree based on the combined ITS-LSU-SSU-TEF1-RPB1 5-gene dataset, using strains from the genus in Magnaporthaceae as the main reference strains and *Cryphonectria parasitica* EP155 as the outgroup. It was found that the ITS alignment contained 584 nucleotides, 870 in LSU, 1034 in SSU, 770 in RPB1, and 926 in TEF1. The 5-gene concatenated dataset comprised 4184 aligned characters, including 796 parsimony-informative sites, 584 variable and parsimony-uninformative sites, and 2804 conserved sites. The best-fit substitution models, determined using jModelTest 2.1.10 and IQ-TREE v.2.3.6, were TIM2+I+G for Bayesian Inference (BI) and TIM2+F+R3 for Maximum Likelihood (ML). The BI and ML phylogenetic trees exhibited congruent topologies; thus, only the BI tree is presented (Figure 1). The entire phylogenetic tree was divided into three distinct branches, A, B, and C, which together constitute the Magnaporthaceae. The strain 1R13 was located in branch C and clustered with *B. longisporus* with a 0.97 BIPP and 51% MLBP, indicating that it belonged to the genus Bussabanomyces in Magnaporthaceae. Additionally, the morphology of strain 1R13 and *B. longisporus* was quite different [17,31].

### 3.2. Morphological Observation

**Figure 2:**
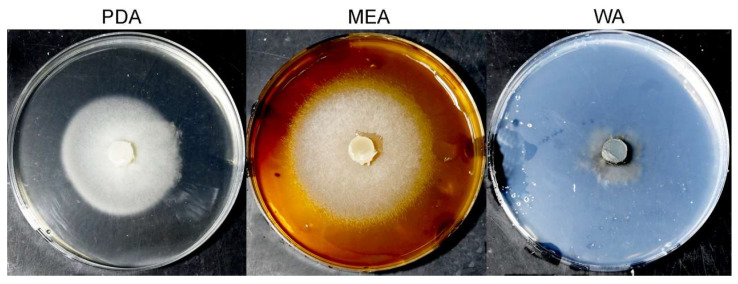
The colony morphology growing on PDA, MEA, and WA media after 5 d of growth. PDA, potato dextrose agar; MEA, malt extract agar; and WA, water agar.

**Figure 3:**
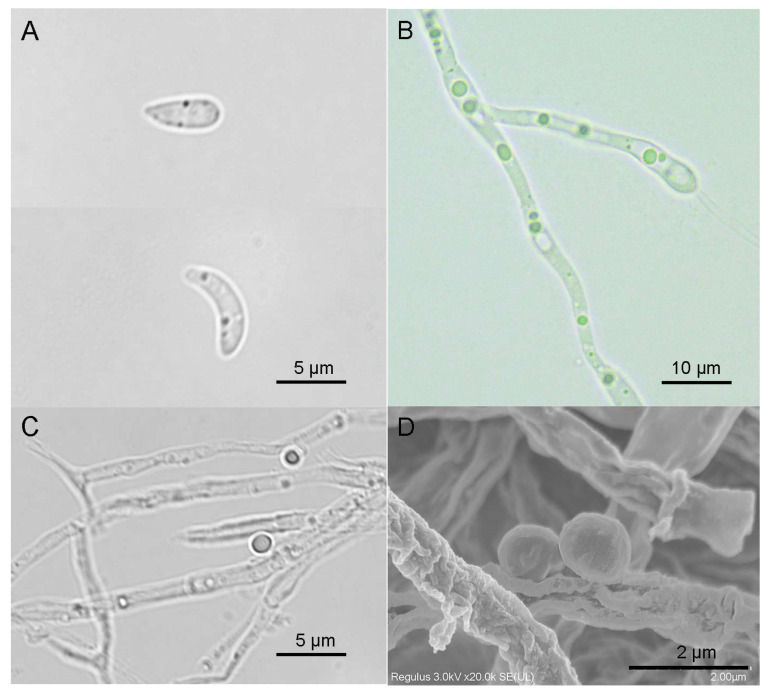
The morphology observation of the hyphae and conidia. (**A**) Two types of conidia germinated in PDB medium. Bar = 5 μm. (**B**) Hyphae germinated in PDB medium. Bar = 10 μm. (**C**) Conidia and hyphae produced in PDA medium. Bar = 5 μm. (**D**) The conidia and hyphae produced by the PDA culture medium were observed under the scanning electron microscope. Bar = 2 μm.

*Bussabanomyces oryzae* nov. (Collection Number: CCTCC M 2025948) (Figure 2 and Figure 3).

*Etymology*: Isolated from *Oryzae sativa*.

*Colonies* on PDA and MEA medium were round with regular edges, and the front side of the colonies was white, reaching 9 cm in 10 d. On WA medium, colonies were dark, reaching 3 cm in 10 d (Figure 2). *Conidia*, one being sickle-shaped and the other being ovoid, in PDB medium (Figure 3A,D). Oval-shaped conidia, 4–6 μm in length and 1.2–1.7 μm in width. Sickle-shaped conidia, 6–10 μm in length and 0.8–1.3 μm in width. In PDA medium, round, with a diameter of approximately 0.5–0.8 μm (Figure 3D). *Hyphae* 1.2–3.8 μm width, no septum, transparent in PDB medium (Figure 3B). Transparent and had septa in PDA medium (Figure 3C).

Based on molecular phylogeny and morphological and biological characteristics, the strain 1R13 was defined as a new species, *Bussabanomyces oyrzae* sp. nov (Collection Number: CCTCC M 2025948).

### 3.3. The Colonization Pattern of Bussabanomyces oryzae in the Roots of Rice

**Figure 4:**
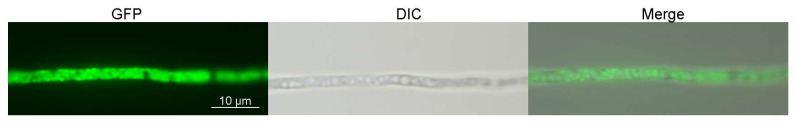
Identification of GFP-labeled fluorescent transformants. The hyphae showed constitutive GFP expression. Bar = 10 μm. GFP, green fluorescent protein; DIC, differential interference contrast; merge, GFP and DIC images are merged together.

The transformants were obtained by Agrobacterium-mediated transformation. Through two rounds of purification, 15 GFP-labeled transformants were selected. Under a fluorescence microscope, a transformant with green fluorescence was picked up for the subsequent rice co-culture assay (Figure 4).

**Figure 5:**
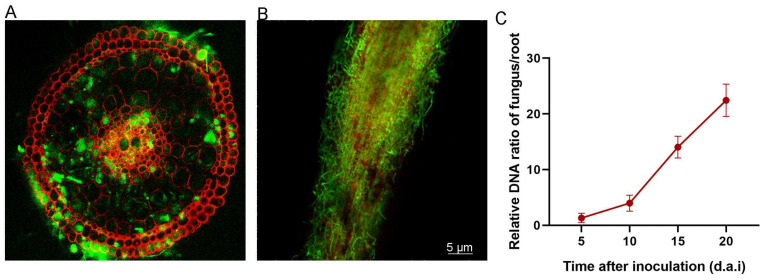
The colonization pattern of *B. oryzae* in rice roots. (**A**) The colonization pattern on the cross-section. The red fluorescent signal represents the root tissue of the rice, while the green fluorescent signal is that of the GFP-labeled hyphae. Bar = 5 μm. (**B**) The colonization pattern on the longitudinal section. The red fluorescent signal represents the root tissue of the rice, while the green fluorescent signal is that of the GFP-labeled hyphae. Bar = 5 μm. (**C**) Relative amounts of fungal/rice DNA at different time points (5, 10, 15, 20 d.a.i).

The roots of the co-cultured rice were cross-cut and longitudinally cut to observe the colonization pattern of *B. oryzae.* Through fluorescence microscopic observation, it was found that a large number of GFP-labeled hyphae were concentrated at the epidermis cells and then invaded the root cells (Figure 5A). Additionally, GFP-labeled hyphae extensively colonized root tissues, progressing from the epidermis through the outer cortex to the inner cortex (Figure 5B).

The infection of *B. oryzae* in the roots of rice can be reflected by determining the relative DNA ratio of fungus/root (FPDR). The results showed that the FPDR of *B. oryzae* was relatively low after co-culture for 5 d; the ratio was only 1.32 ± 0.83. Then it increases gradually. During the period of co-cultivation with rice for 10 to 15 d, the growth rate was the fastest and reached the maximum at 20 d (Figure 5C).

### 3.4. Bussabanomyces oryzae Promote the Growth of Rice

**Figure 6:**
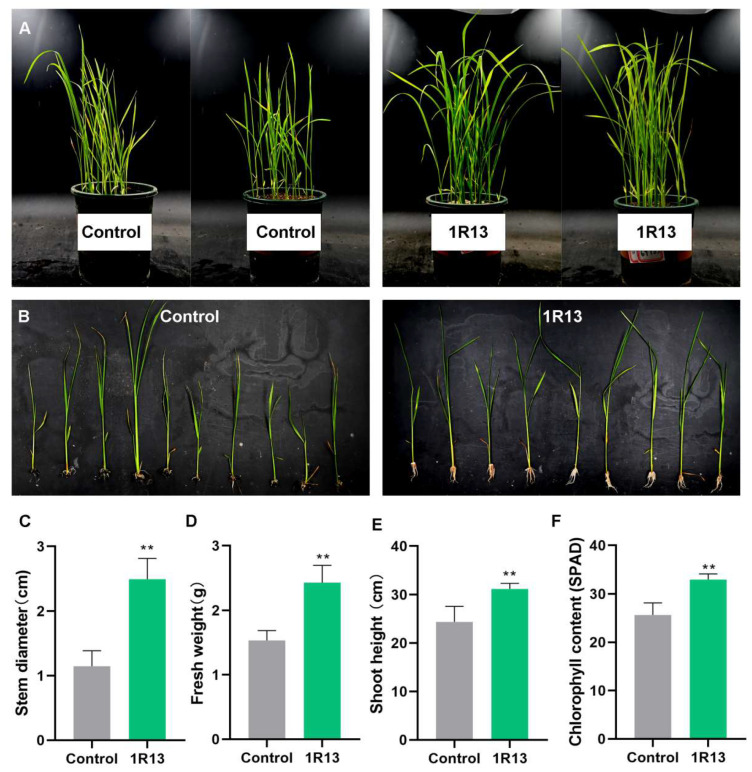
The phenotype of co-cultivated rice. (**A**,**B**) Rice plants after co-cultivation with *B. oryzae* for 14 d. (**C**–**F**) The comparison of phenotype between the rice plants treated with *B. oryzae* and the non-treated control included stem diameter (**C**), fresh weight (**D**), shoot height (**E**), and chlorophyll content (**F**). All the bar charts were plotted with the mean ± standard deviation. Independent sample *t*-tests were used to analyze the data. All treatments were repeated three times, with 50 rice plants being measured in each treatment, and three independent experimental repetitions were conducted. The symbol ** indicates a significant difference at *p* < 0.01.

The fermentation broth of 1R13 was prepared and added to the rice seedlings. The phenotype of the rice seedlings was observed after 15 d (Figure 6A,B). It could be seen from the results that *B. oryzae* significantly promoted the growth of rice seedlings. The shoot height of the rice seedlings with 1R13 fermentation broth added was approximately 6 cm higher than that of the control (Figure 6E). Meanwhile, the rice with 1R13 fermentation broth added appeared stronger than the control (Figure 6C). Therefore, the fresh weight of rice in the treatment was significantly higher than that in the control. In addition, *B. oryzae* also increased the chlorophyll content of rice and might play an important role in promoting photosynthesis in rice (Figure 6F).

### 3.5. Bussabanomyces oryzae Enhances the Resistance of Rice to Rice Blast

**Figure 7:**
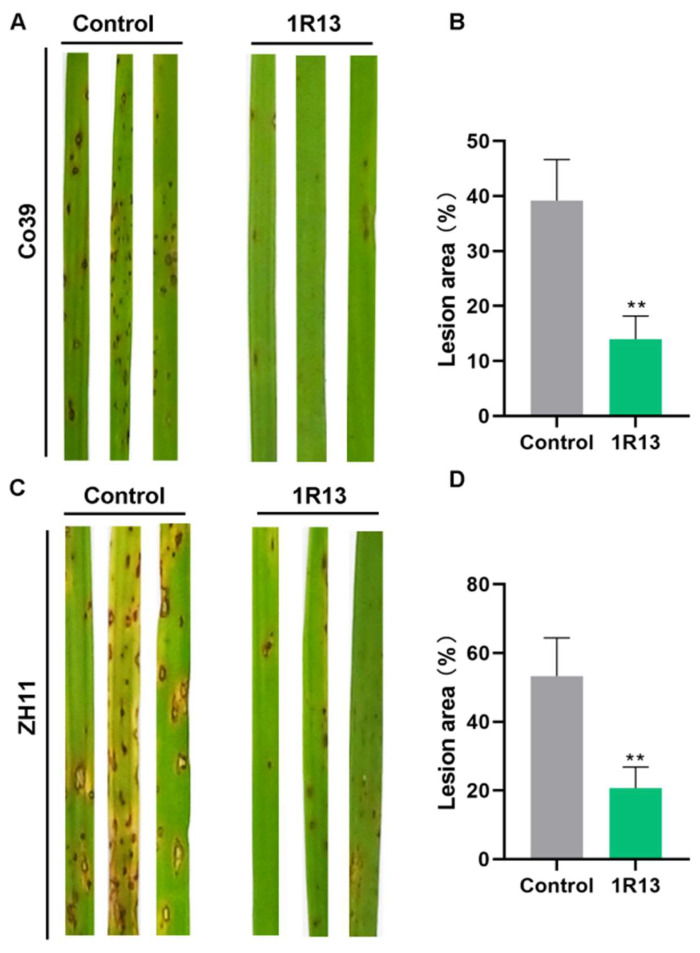
The effect of *B. oryzae* on rice resistance. The degree of severe symptomatic damage and lesion area manifested on the foliage of CO39 rice variety (**A**,**B**) and ZH11 rice variety (**C**,**D**) co-cultivated with *B. oryzae.* The rice plants that were treated with the ordinary fermentation broth were used as the control. All the bar charts were plotted with the mean ± standard deviation. The data were analyzed by independent sample *t*-tests. All treatments were repeated three times, with 20 rice leaves being measured in each treatment, and three independent experimental repetitions were conducted. The symbol ** indicates a significant difference at *p* < 0.01.

The effect of *B. oryzae* on rice resistance was determined by conducting a spray assay of rice blast fungus on rice seedlings. The results showed that *B. oryzae* could enhance the resistance of different varieties of rice to rice blast (Figure 7A,C). Among the indica rice CO39, the rice treated with 1R13 had fewer disease spots in leaves, with the lesion area being only 14%, which was much lower than 40% in the control (Figure 7B). In japonica rice ZH11, when the occurrence of rice blast was relatively severe, 1R13 could also significantly enhance the resistance of rice to rice blast, reducing the lesion area by about 30% (Figure 7D).

### 3.6. The Influence of Bussabanomyces oryzae on Rice Seedlings in Fields

**Figure 8:**
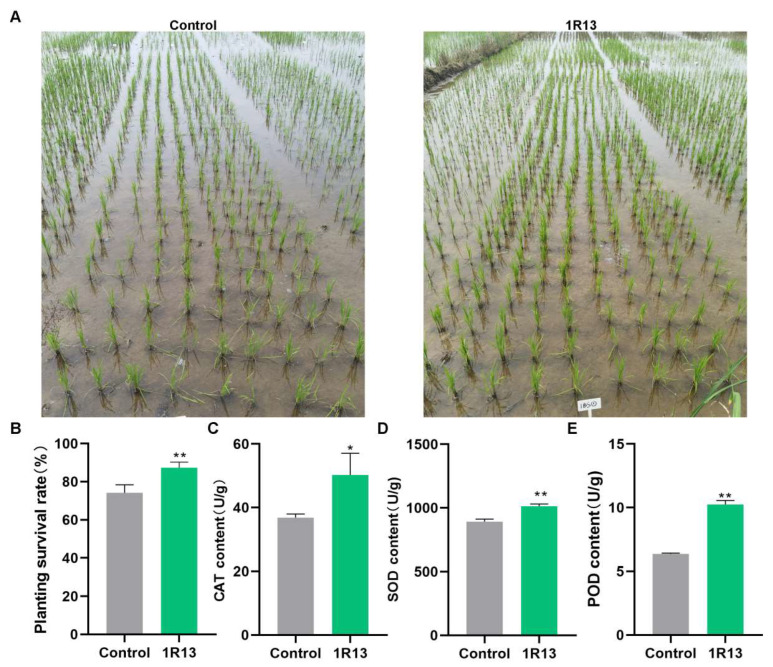
The phenotype of rice seedlings in fields. (**A**) The growth condition of the rice seedlings after transplantation to the field 15 d after *B. oryzae* treatment. The rice plants that were added with the ordinary fermentation broth were used as the control. (**B**–**E**) The comparison of phenotype between the rice plants treated with *B. oryzae* and the control included planting survival rate (**B**), CAT content (**C**), SOD content (**D**), and POD content (**E**). All the bar charts were plotted with the mean ± standard deviation. Independent sample *t*-tests were used to analyze the data. All treatments were repeated three times, with 100 rice plants being measured in each treatment, and three independent experimental repetitions were conducted. The symbols * and ** indicate significant differences at *p* < 0.05 and *p* < 0.01, respectively.

The rice seedlings treated with 1R13 were transplanted into the field to observe its effect on the seedlings. The results showed that 15 d after transplanting the seedlings, the survival rate of the seedlings treated with *B. oryzae* was about 87.4%, which was 13% higher than that of the control (Figure 8A,B). By determining the antioxidant enzyme activities of the seedlings, it was found that the content of CAT, SOD, and POD of the seedlings treated with *B. oryzae* was significantly higher than that of the control (Figure 8C–E). *B. oryzae* enhances the adaptability of rice to adverse conditions by increasing the activity of antioxidant enzymes.

### 3.7. The Expression of Defense-Related Genes in Rice

**Figure 9:**
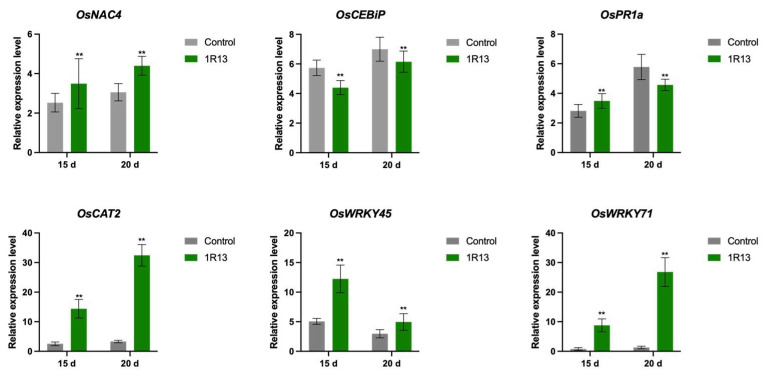
The relative expression levels of defense-related genes in *B. oryzae*-treated and control-treated rice plants. The relative expression levels of defense-related genes in *B. oryzae*-treated rice plants after 15 d and 20 d were calculated using the mean ± standard deviation method. Independent sample *t*-tests were used to analyze the data. All treatments were repeated three times, with 50 rice plants being measured in each treatment, and three independent experimental repetitions were conducted. The symbol ** indicates a significant difference at *p* < 0.01, respectively.

We analyzed the relative expression of defense-related genes in rice, including the pathogenesis-related gene (PR) *PR1a*, the reactive oxygen-related gene *CAT2*, the transcription factor *WRKY45*, and the immune reaction-related gene *CEBiP*. The results showed that at 15 d of *B. oryzae* treatment, the *CEBiP* gene was significantly downregulated, while the expression of other genes was significantly upregulated. When treated for 20 d, the *CEBiP* gene was still downregulated, and the *PR1a* gene, which was previously upregulated, was also downregulated (Figure 9). The mean difference, standard error, and t values are listed in Supplementary Table S3. These results indicate that the invasion of *B. oryzae* could regulate the differentiated expression of some defense-related genes, thereby enhancing the stress resistance of rice.

## 4. Discussion

Rice (*Oryza sativa*), a staple crop for a significant portion of the global population, is constantly threatened by various diseases, most notably rice blast caused by *M. oryzae*, leading to substantial yield losses [32]. Current management strategies often rely on chemical fungicides, which pose environmental and health risks [33]. Therefore, exploring sustainable and eco-friendly alternatives, such as the use of beneficial endophytic fungi, is of paramount importance for enhancing crop resilience and productivity. This study identified a novel endophytic fungus, *Bussabanomyces oryzae*, from rice roots and demonstrated its potential to promote growth and induce resistance against rice blast, offering a promising biological resource for sustainable rice cultivation.

The key finding of this study was the identification of strain 1R13 as a novel species within the Magnaporthaceae family. While the genus Bussabanomyces has been previously described, phylogenetic analysis clearly placed 1R13 as a distinct singleton, separate from *B. longisporus* (Figure 1). Furthermore, there are also significant differences between the morphology of conidia and hyphae. Thel conidia of *B. longisporus* are obclavate in shape, with four to five septa, measuring 47–72 μm (length) × 5.6–7.6 μm (width). The hyphae are mostly in an irregular shape [31]. The conidia produced by 1R13 mainly fall into two types, one being sickle-shaped and the other being ovoid (Figure 3A,D). This type of sporulation pattern has also been observed in other studies of endophyte [30]. However, the significance of this sporulation pattern still requires further investigation.

In our study, we found that the hyphae of *B. oryzae* first accumulated on the epidermal cells of the rice roots and gradually invaded the cortex and outer cortex of the root cells. The hyphae finally reached the vascular bundle cells (Figure 5A). This colonization pattern was consistent with that of most endophytic fungi [34]. However, a notable point of divergence in our study was the absence of specialized infection structures like appressoria, which are commonly employed by pathogenic and some endophytic fungi to breach the host cell wall [35]. Instead, *B. oryzae* appeared to rely on direct hyphal growth for invasion. This mode of colonization, coupled with the lack of any visible damage or lesion formation in the host, strongly aligns with the characteristics of dark septate endophytes (DSEs), a functional group of root-colonizing fungi [36]. Meanwhile, the infection rate of *B. oryzae* reached its peak mainly during the period of co-cultivation with rice for 10 to 15 d. And the FPDR continued to increase within the 20-day period (Figure 5B). This colonization pattern further supports the establishment of a compatible and long-term symbiotic relationship. We hypothesize that, similar to other DSEs [37], *B. oryzae* may secrete effector proteins or other compounds that modulate the host’s immune responses, allowing for accommodation without triggering strong defense reactions, a key aspect of a successful mutualistic symbiosis.

The growth-promoting effect of *B. oryzae* on rice, evidenced by increased fresh weight and chlorophyll content (Figure 6D–F), corroborates the well-documented beneficial roles of endophytes in enhancing plant productivity [38]. This finding is consistent with numerous studies on rice–endophyte interactions. *Phialemonium dimorphosphorum* demonstrated the capability to promote the growth of rice seedlings in terms of seed germination, plant height, root length and degree of root colonization [13]. *Absidia* sp. and *Cylindrocladium* sp. showed significant increases (*p* ≤ 0.05) in plant height and high growth inhibition of the pathogen *M. oryzae* [39]. However, the mechanism by which these endophytic fungi promoted the growth of rice had not yet been studied. Zhu et al. [15] demonstrated that the rice endophyte *Pseudophialophora oryzae* promotes host growth by upregulating genes involved in nutrient uptake (N, P, K, Fe, Mg). While our study did not directly assay for phytohormone production, the observed increase in chlorophyll content suggests a plausible mechanism. Higher chlorophyll levels are directly linked to enhanced photosynthetic capacity, which could, in turn, fuel increased biomass accumulation. Future work should investigate whether *B. oryzae* employs similar strategies, such as phytohormone production, nutrient mobilization, or photosynthetic enhancement, to promote rice growth.

During the interaction between endophytes and plants, the most important role of these fungi was their ability to help the host resist adverse environments. Endophytes are increasingly recognized for their ability to prime the host immune system for a faster and stronger response to challenges, a phenomenon known as induced systemic resistance (ISR) [40]. *P. indica* enhanced the immune capacity of plants and improved immune resistance by increasing the activity of host protective enzymes and inducing the expression of disease-resistant related genes such as PRRs [41,42]. *Trichoderma harzianum* upregulates the expression of pathogenesis-related genes and enzymes to enhance gray blight resistance in tea [43]. In our results, *B. oryzae* promotes host resistance by increasing the activity of host protective enzymes and inducing the expression of disease-resistance-related genes (Figure 8 and Figure 9). The results indicate that *B. oryzae* could directly stimulate the expression of the *OsNAC* and *OsPR1a genes* in rice, which were crucial mediators of induced systemic resistance [44,45]. Interestingly, we observed a continuous decrease in OsCEBiP expression (Figure 9). OsCEBiP, a chitin receptor gene, is involved in chitin perception, which is typically the first step in fungal recognition [46]. It is possible that *B. oryzae* actively suppresses this early recognition step to facilitate colonization—a strategy observed in some mutualistic interactions, such as the suppression of basal defense by *P. indica* during early colonization [47]. *OsWRKY71* and *OsWRKY45* are associated with the salicylic acid (SA) and jasmonic acid (JA) signaling pathways, respectively [48]. These pathways are the main regulatory mechanisms for inducing systemic resistance in rice [49]. The expression levels of the *OsWRKY71* and *OsWRKY45* genes were upregulated by inoculating *B. oryzae* (Figure 9), indicating that the systemic resistance of *B. oryzae* to rice blast might be mediated jointly by the salicylic acid and jasmonic acid pathways.

## 5. Conclusions

We isolated an endophyte 1R13 from the roots of rice. Through morphological observation and phylogenetic identification, we defined it as *Bussabanomyces oryzae.* It was proposed to be a new species, *Bussabanomyces oryzae* nov. Through co-culture with rice, it was found that *B. oryzae* mainly invaded through the epidermal cells of the roots and entered the cortical and vascular bundle cells of rice. Further research has shown that *B. oryzae* could promote the growth of rice, increase its fresh weight and chlorophyll content, and enhance the resistance of rice to adverse environments and rice blast disease. Through the qRT-PCR assay, it was found that *B. oryzae* mainly exerted the host’s stress resistance by regulating the expression of some defense-related genes. The discovery of *B. oryzae* as a novel species not only expands the current understanding of fungal diversity associated with rice but also provides a valuable microbial resource for agricultural biotechnology. However, key challenges remain, including understanding its host specificity, long-term ecological impact, and the optimization of its formulation for large-scale agricultural use. Addressing these questions will be essential for translating this laboratory-based discovery into practical strategies for enhancing global food security.

## Figures and Tables

**Table 1:** Species name, isolated ID, and GenBank accession numbers of the fungi used in this study.

Species Name	Isolate ID	18S(SSU)	ITS	28S(LSU)	RPB1	TEF1
*Bussabanomyces oyrzae*	1R13	PV848745	PV848743	PV848744	PV855959	PV855960
*Buergenerula spartinae*	ATCC22848	DQ341471	JX134666	DQ341492	JX134720	JX134692
*Bussabanomyces longisporus*	CBS 125232	KM009214	KM009166	KM009154	KM009190	KM009202
*Gaeumannomyces graminis* var. avenae	CBS187.65	JX134655	JX134668	JX134680	JX134722	JX134694
*Gaeumannomyces graminis* var. graminis	M33	JF414871	JF710374	JF414896	JF710442	JF710411
*Gaeumannomyces graminis* var. graminis	M54	JF414873	JF414848	JF414898	JF710444	JF710419
*Gaeumannomyces graminis* var. tritici	M55	JF414875	JF414850	JF414900	JF710445	JF710420
*Kohlmeyeriopsis medullaris*	JK5522N	/	KM484853	KM484969	KM485069	/
*Kohlmeyeriopsis medullaris*	JK5528S	/	KM484852	KM484968	KM485068	/
*Magnaporthiopsis agrostidis*	BRIP 59300	MF178145	KT364753	KT364754	KT364755	KT364756
*Magnaporthiopsis cynodontis*	D29387-3	MK458746	MK458730	MK458740	MK458761	MK458756
*Magnaporthiopsis incrustans*	M35	JF414867	JF414843	JF414892	JF710437	JF710412
*Magnaporthiopsis incrustans*	M51	JF414870	JF414846	JF414895	JF710440	JF710417
*Magnaporthiopsis maydis*	M84	KM009208	KM009160	KM009148	KM009184	KM009196
*Magnaporthiopsis maydis*	M85	KM009209	KM009161	KM009149	KM009185	KM009197
*Magnaporthiopsis meyeri*-festucae	FF2	MF178140	MF178146	MF178151	MF178162	MF178167
*Magnaporthiopsis panicorum*	CM2s8	KF689593	KF689643	KF689633	KF689613	KF689623
*Magnaporthiopsis poae*	M47	JF414860	JF414836	JF414885	JF710433	JF710415
*Magnaporthiopsis rhizophila*	M23	JF414858	JF414834	JF414883	JF710432	JF710408
*Nakataea oryzae*	M21	JF414862	JF414838	JF414887	JF710441	JF710406
*Omnidemptus affinis*	ATCC 200212	JX134660	JX134674	JX134686	JX134728	JX134700
*Ophioceras commune*	M91	JX134661	JX134675	JX134687	JX134729	JX134701
*Pseudophialophora eragrostis*	CM12m9	KF689598	KF689648	KF689638	KF689618	KF689628
*Pseudophialophora eragrostis*	CM20m5-2	KF689597	KF689647	KF689637	KF689617	KF689627
*Pseudophialophora panicorum*	CM3m7	KF689602	KF689652	KF689642	KF689622	KF689632
*Pseudophialophora panicorum*	CM9s6	KF689601	KF689651	KF689641	KF689621	KF689631
*Pseudophialophora schizachyrii*	AL2m1	KF689599	KF689649	KF689639	KF689619	KF689629
*Pseudophialophora schizachyrii*	AL3s4	KF689600	F689650	KF689640	KF689620	KF689630
*Pyricularia grisea*	M82	JX134656	JX134670	JX134682	JX134724	JX134696
*Slopeiomyces cylindrosporus*	CBS 610.75	DQ341473	JX134667	DQ341494	JX134721	JX134693
*Cryphonectria parasitica*	EP155	Genome data, GCF_011745365				

## Data Availability

The data presented in this study are openly available in Genebank at https://account.ncbi.nlm.nih.gov/?back_url=https%3A%2F%2Fwww%2Encbi%2Enlm%2Enih%2Egov%2FWebSub%2F%3Fform%3Dhistory%26tool%3Dgenbank.

## Supplementary Materials

The following supporting information can be downloaded at: https://www.mdpi.com/article/10.3390/jof12030222/s1, Table S1: Primers mentioned in the article; Table S2: The BLAST results of the five gene sequences of 1R13; Table S3: The mean difference, standard error, and t value of defense-related genes in *B. oryzae*-treated and control-treated rice plants.
