# Supplementary material for: A New Species Bussabanomyces oryzae Isolated from Rice and Beneficial Application in Rice Seedling
Source: J Fungi (Basel). 2026 Mar 19;12(3):222. doi: 10.3390/jof12030222 (PMC13027448; doi:10.3390/jof12030222)
Supplement: Supplementary file 1 [file jof-12-00222-s001.zip › jof-4190107-supplementary tables.pdf]

Table S1 Primers mentioned in the article.

Primer name	Primer sequence (5’-3’)
ITS1	TCCGTAGGTGAACCTGCGG
ITS4	TCCTCCGCTTATTGATATGC
RPB1-F	GARTGYCCDGGDCAYTTYGG
RPB1-R	CCNGCDATNTCRTTRTCCATRTA
TEF1-F	GCYCCYGGHCAYCGTGAYTT
TEF1-R	ATGACACCRACRGCRACRGTYTGYAT
LSU-F	GTACCCGCTGAACTTAAGC
LSU-R	TCCTGAGGGAAACTTCG
SSU-F	GTAGTCATATGCTTGTCTC
SSU-R	CTTCCGTCAATTCCTTTAAG
OsUbiq-F	GTGGTGGCCAGTAAGTCCTC
OsUbiq-R	GGACACAATGATTAGGGATCA
NAC4-F	AAGCGCAGCATCAACAAAG
NAC4-R	TCCATCCTTCTCCTCTCGTG
CEBiP-F	GATGACTGGTTTATCCAGCTTTG
CEBiP-R	TTCAAGCAGCCGTACAAGTG
PR1a-F	TCTTCATCACCTGCAACTACTC
PR1a-R	ATTCATCGGATTTATTCTCACC
CAT2-F	TAATAGTAATACGTGCTA
CAT2-R	GTGATGTCGGTGACGTCGTGGG
OsWRKY45-F	CGGGTAAAACGATCGAAAGA
OsWRKY45-R	TTTCGAAAGCGGAAGAACAG
OsWRKY71-F	AGATGGCGATGACGCTGAC
OsWRKY71-R	AGCAATCGTCAATCCTTGGT

Table S2 The BLAST results of the five gene sequences of 1R13.

Gene	Strain	Accession	Identity
ITS	Magnaporthaceae sp. isolate ND00J18	ON063283.1	99.61%
LSU	Magnaporthaceae sp. HP24-3	KM401637.1	99.31%
SSU	Pseudophialophora sp. isolate LJJ23	MT379655.1	98.83%
RPB1	Magnaporthaceae sp. JG-2022a	OM938229.1	93.43%
TEF1	Magnaporthaceae sp. HP24-3	KP282703.1	95.50%

Table S3 The mean difference, standard error, and t value of defense-related genes in *B.oryzae*-treated and control-treated rice plants.

Gene	Time point	Mean difference	Standard error	t value	p-value
OsNAC4	15 d	0.96	0.191	5.03	< 0.01
OsNAC4	20 d	1.343	0.0918	14.64	< 0.01
OsCEBiP	15 d	1.33	0.100	13.3	< 0.01
OsCEBiP	20 d	0.84	0.153	5.49	< 0.01
OsPR1a	15 d	0.67	0.092	7.28	< 0.01
OsPR1a	20 d	1.21	0.133	9.10	< 0.01
